# Antibiotic resistance and molecular characterization of diarrheagenic *Escherichia coli* and non-typhoidal *Salmonella* strains isolated from infections in Southwest China

**DOI:** 10.1186/s40249-018-0427-2

**Published:** 2018-05-07

**Authors:** Shun-Xian Zhang, Yong-Ming Zhou, Li-Guang Tian, Jia-Xu Chen, Rita Tinoco-Torres, Emmanuel Serrano, Shi-Zhu Li, Shao-Hong Chen, Lin Ai, Jun-Hu Chen, Shang Xia, Yan Lu, Shan Lv, Xue-Jiao Teng, Wen Xu, Wen-Peng Gu, Si-Tang Gong, Xiao-Nong Zhou, Lan-Lan Geng, Wei Hu

**Affiliations:** 1 Guangzhou Institute of Pediatrics, Guangzhou Women and Children’s Medical Center, Guangzhou Medical University, Guangzhou, 510623 People’s Republic of China; 2 Department of Gastroenterology, Guangzhou Women and Children’s Medical Center, Guangzhou Medical University, Guangzhou, 510623 People’s Republic of China; 3 Yunnan Provincial Center for Disease Control and Prevention, Kunming, 650022 People’s Republic of China; 4 National Institute of Parasitic Diseases, Chinese Center for Disease Control and Prevention, Shanghai, 200025 People’s Republic of China; 5 Chinese Center for Tropical Diseases Research; WHO Collaborating Centre for Tropical Diseases; National Center for International Research on Tropical Diseases, Ministry of Science and Technology; Key Laboratory of Parasite and Vector Biology, Ministry of Health, Shanghai, 200025 People’s Republic of China; 6 Department of Biology & CESAM, University of Aveiro, 3810-193 Aveiro, Portugal; 7 Wildlife Ecology and Health group and Servei d’Ecopatologia de Fauna Salvatge (SEFaS), Departament de Medicina i Cirurgia Animals, Universitat Autònoma de Barcelona (UAB), Bellaterra, Spain; 8 Department of Microbiology and Microbial Engineering, School of Life Sciences, Fudan University, Shanghai, 200433 People’s Republic of China

**Keywords:** Enterobacterial infections, Gastroenteritis, Fingerprint typing, Kunming, Yunnan

## Abstract

**Background:**

Bacterial diarrhea is one of the most common causes for medical consultations, mortality and morbidity in the world. Diarrheagenic *Escherichia coli* (DEC) and non-typhoidal *Salmonella* (NTS) are major intestinal pathogens in developing countries, and the indiscriminate use of antibiotics has greatly contributed to resistant strains. Hence, the aim of the present study is to identify the antimicrobial resistance patterns and the molecular characteristics of DEC and NTS in southwest, China.

**Methods:**

1121 diarrheal patients and 319 non-diarrheal subjects across all age groups were recruited from four sentinel hospitals from June 2014 to July 2015 in Kunming City, Yunnan Province. Each stool specimen was collected to detect DEC and NTS with standard microbiological and molecular methods. Antimicrobial resistance testing was performed with the Kirby-Bauer disk diffusion method, and the standards for antimicrobial susceptibility testing complied with the Clinical and Laboratory Standards Institute (CLSI). Molecular characterization of strains was carried out using pulsed-field gel electrophoresis (PFGE). A structured questionnaire was used to record basic epidemiological data (e.g. sex, age, residence, season, etc.). Data were analyzed using Chi-square or Fisher’s exact test.

**Results:**

DEC was detected in 127 (11.33%) diarrhea cases and 9 (2.82%) non-diarrheal cases (*χ*^2^ = 20.69, *P* < 0.001, *OR* = 4.36, 95% *CI*: 2.19–8.65), and the prevalence of NTS isolated from diarrhea cases was higher than that of non-diarrheal cases across all age groups (*n* = 42, 3.75%, *n* = 1, 0.31%, *χ*^2^ = 10.10, *P* = 0.002, *OR* = 12.38, 95% *CI*: 1.70–90.29). The rates of resistance to ten antibiotics of DEC and NTS showed significant differences (*χ*^*2*^ = 386.77, *P* < 0.001; *χ*^2^ = 191.16, *P* < 0.001). The rates of resistance to Amoxicillin and Clavulafiate (AMC), Cephalothin (CEP), Gentamicin (GEN) and Sulfamethoxazole-Trimethoprim (SXT) of DEC isolated from diarrhea cases were higher than those of NTS isolated from diarrhea patients (37.01% vs 14.29%, *χ*^2^ = 7.57, *P* = 0.006; 29.92% vs 11.90%, *χ*^2^ = 5.40, *P* = 0.02; 37.01% vs 11.90%, *χ*^2^ = 5.80, *P* = 0.016; 62.20% vs 26.19%, *χ*^2^ = 16.44, *P* < 0.001; respectively). Ciprofloxacin (CIP) was the most sensitive antibiotic for DEC and NTS strains isolated from diarrhea cases. Resistance rates of DEC isolates from cases and controls to more than three kinds antimicrobials (multidrug resistance, MDR) showed no significant differences (81.10% vs 88.89%, *P* = 0.33). Pulsotype patterns of DEC strains were highly diverse; however, the pulsotype pattern of NTS strains was closely related to the serotype. The pattern of *S. enteritidis* was highly similar, but the *S. enterica* Typhimurium strain was discrete.

**Conclusions:**

Antibiotic resistance of *Enterobacteriaceae* is of great concern. The societal effects of antibiotic use justify strict monitoring to combat increases in antimicrobial resistance. Molecular epidemiology and systematic epidemiological investigation can provide accurate evidence for tracking the infection source.

**Electronic supplementary material:**

The online version of this article (10.1186/s40249-018-0427-2) contains supplementary material, which is available to authorized users.

## Multilingual abstract

Please see Additional file 1 for Translation of the abstract into the five official working languages of the United Nations.

## Background

Acute diarrheal illness is still a major public health problems resulting in medical consultations, mortality and morbidity worldwide, especially in low- and middle-income countries [1–3]. In addition, diarrhea disease is one of the leading threats to children’s health, with 2.8 billion episodes and recent statistics indicating 700 000 deaths worldwide per year in children under the age of five [1, 4]. Globally, 2.7 diarrheal episodes are estimated to occur in every child (< 5 years) [1].

Enteric bacterial pathogens, and their products, are the major causes of acute diarrhea [4–7], including diarrhoeagenic *Escherichia coli* (DEC), non-typhoidal *Salmonella* (NTS), *Shigella* spp. and *Vibrio cholerae*, among others. In China, annually, 70 million cases of infectious diarrhea are reported [8], but in only 5.0% of cases is a pathogen identified, which may be due to a lack of appropriate technology and funding [8, 9]. Thus, indiscriminate antibiotic treatment is crucial for weak individuals with severe bacterial infection. For example, cefotaxime (CTX) has been applied to treat infectious illnesses involving gram-negative bacteria in recent years in China [10]. However, the abuse of antibiotics increases the selection pressure for resistant strains and decreases the effectiveness of antibiotics [11, 12]. In addition, multi-drug resistance (MDR) strains and the extended-spectrum β-lactamases (ESBL)-producing strains are increasingly reported in humans and animals [13–16]. Moreover, the prevalence of *Enterobacteriaceae* producing carbapenemases increased in recent years, which results in a treatment impasse and challenges to the treatment of diarrheal illness [17].

Pulsed-field gel electrophoresis (PFGE) has become an important tool in solving public health problems in many countries in recent years [18–20]. It is considered a gold standard method with the advantage of accuracy and reliability. Hence, it was applied here to identify and trace DEC and NTS strains [21].

DEC and NTS strains were isolated from diarrhea and non-diarrhea subjects in southwest China [9, 22]. The aim of present study was to identify the antimicrobial resistance patterns and the molecular characteristics of DEC and NTS. For this purpose, we compared diarrheal patients and non-diarrheal subjects across all age groups, from different hospitals in Kunming City, Yunnan Province, China. We discuss our results in light of their utility as a reference for the treatment and prevention of diarrhea illness associated with bacterial causative agents.

## Methods

### Subjects of this study

Acute gastroenteritis patients were defined as those who had diarrhea more than three times within a 24 h period, with abnormal stool specimens (e.g. mucus in the stool, watery stool, loose stool or bloody stool) in accordance with the WHO standard [23]. Non-diarrheal subjects were defined as those who had no history of diarrhea symptoms in the 14 days prior to recruitment into the present study, and were recruited at the same time as the diarrheal subjects.

### Specimen and data collection

A non-matched case-control study was designed and conducted. Each stool specimen was collected from each subject across all age groups (including diarrheal patients and non-diarrheal cases) in outpatients from four sentinel hospitals: i) The First People’s Hospital of Yunnan Province, ii) The Kunming Children’s Hospital, iii) The Pushan Community Health Service Center in Kunming, and iv) The First Affiliated Hospital of Kunming Medical University. A sterile plastic sampling cup was used to collect stool samples, with the criterion that each stool sample must be greater than 3 g or 3 ml. Each sample was delivered to the laboratory of Yunnan Provincial Center for Disease Control and Prevention in Cary-Blair (C-B) culture medium (Oxoid Ltd., Basingstoke, UK) within 12 h. Basic epidemiological information (e.g. sex, age, residence, season, etc.) was collected with a structured questionnaire by doctors or nurses. The study was conducted from July 2014 to June 2015.

### Laboratory tests for DEC and NTS

The DEC strain was divided into five subtypes by their virulence genes as follows: enteroaggregative *E. coli* (EAEC), enterotoxigenic *E. coli* (ETEC), enteropathogenic *E. coli* (EPEC), enteroinvasive *E. coli* (EIEC) and enterohaemorrhagic *E. coli* (EHEC). Each stool specimen was inoculated in MacConkey agar (MAC, Oxoid Ltd., Basingstoke, UK) and cultured at 37 °C for 18 h. Ten putative DEC colonies were then selected and mixed with 150 μl water to extract DNA at 100 °C for 10 min. The 20 μl volume of quantitative PCR (qPCR) mix was composed of 10 μl master mix (Takara Bio Inc., Shlga, Japan), 1 μl forward primer (10 μmol)[9, 22], 1 μl reverse primer (10 μmol), 1 μl DNA template and 7 μl H_2_O. The cycling conditions for each subtype DEC were 95 °C for 5 min, 40 cycles of 95 °C for 5 s, 60 °C for 30 s. Fluorescence was recorded at the annealing stage. In addition, these ten putative DEC colonies were also inoculated in nutrient agar media to culture a single strain at 37 °C for 18 h and to obtain single colonies. If the qPCR of the putative DEC colony was positive, a colony from the single strains was chosen and DNA extracted. Single primes were selected to detect the subtype of DEC [9]. In addition, each stool sample was inoculated in selenite brilliant green sulfa enrichment broth (SBG, Oxoid Ltd., Basingstoke, UK) for enrichment, and then inoculated in *Salmonella-Shigella* agar (SS, Oxoid Ltd., Basingstoke, UK) to detect NTS. Systematic biochemical identification of NTS was performed using the VITEK® 2 Compact instrument (bioMerieux, Marcyl’Etoile, France). When NTS was determined to be positive, further serological testing was used to identify the subtype. Detailed detection procedures can be found in the references [9, 22, 24, 25].

### Susceptibility testing of enteric bacterial pathogens

The Kirby-Bauer disk diffusion method was used to detect antibiotic susceptibility of enteric bacterial pathogens. These antimicrobials were ampicillin (AMP, 30 μg), amoxicillin/clavulanic acid (AMC, 20/10, 10 μg), cefotaxime (30 μg), cephalothin (CEP, 30 μg), gentamicin (GEN, 10 μg), nalidixic acid (NAL, 30 μg), tetracycline (TCY, 10 μg), ciprofloxacin (CIP, 5 μg), rifampicin (REP, 5 μg) and Sulfamethoxazole-Trimethoprim (SXT, 25 μg). DEC and NTS were cultured at 37 °C for 18–24 h, and the ring size was measured to judge the antibiotic resistance according to the Clinical and Laboratory Standards Institute (CLSI, 2013) of the United States (Table 1). MDR was defined as bacteria pathogens resistant to more than any three commonly used antibiotics according to CLSI. *E. coli* ATCC 25922 was selected as the control strain.

**Table 1:** Performance standards for diarrheagenic *Escherichia coli* and non-typhoidal *Salmonella* to antimicrobial susceptibility testing conducted in the study

Antibiotics	Resistant (cm)	Intermediate (cm)	Susceptible (cm)
AMP	≤13	14–16	≥17
AMC	≤13	14–17	≥18
CEP	≤14	15–17	≥18
CTX	≤22	23–25	≥26
GEN	≤13	13–14	≥15
NAL	≤13	14–18	≥19
CIP	≤11	16–20	≥21
TCY	≤12	12–14	≥15
REP	≤14	15–16	≥17
SXT	≤10	11–15	≥16

Notes: 1: *AMP* Ampicillin, *AMC* Amoxicillin and Clavulafiate, *CEP* Cephalothin, *CTX* Cefotaxime, *GEN* Gentamicin, *NAL* Nalidixic acid, *CIP* Ciprofloxacin, *TCY* Tetracycline, *REP* Rifampicin, *SXT* Sulfamethoxazole–Trimethoprim.2: Performance standards for antimicrobial susceptibility testing conducted in the study was complied with the twenty–third informational supplement from clinical and laboratory standards institute (CLSI). 3: Centimeter (cm), Resistant (R), Intermediate (I), Susceptible (S)

### PFGE

PFGE was conducted to assess clonal-relatedness in accordance with the PulseNet protocol procedure for NTS and *E.coli,* except the O157 serotype. The NTS serotype H9812 was applied as a marker. Agarose-embedded DNA of NTS and DEC was digested with XbaI (Takara Bio Inc., Shlga, Japan). The digestion condition of each plug was 45 U XbaI at 37 °C for 2 h. The CHEF - Mapper (Bio-Rad Laboratories, Inc., Hercules, USA) was used for electrophoresis, and electrophoresis conditions for DEC and NTS were 6.76 s–35.38 s and 2.16 s–63.80 s for 19 h, respectively. A Bio-Rad Gel Doc XR system (Bio-Rad Laboratories, Inc., Hercules, USA) was used to observe and record the gel results. Detailed detection procedures are found in previous studies [26].

### Data analysis

Data analysis was performed by Statistical Product and Service Solutions (SPSS v24.0) software (IBM, US). Odds ratio (*OR*) and 95% confidence intervals (*CI*s) of categorical variables were calculated using two-tailed Chi-square or Fisher’s exact tests. Significant differences were taken as the level of *P* < 0.05 for two-tailed tests. The PFGE patterns of DEC and NTS were analyzed with BioNumerics 5.10 software (Applied Maths, Sint-Martens-Latem, Belgium). A dendrogram was constructed using the Dice similarity coefficient with 1.00% optimization and a tolerance coefficient and un-weighted pair group methods with the arithmetic mean algorithm (UPGMA).

## Results

### The prevalence of DEC and NTS in diarrheal patients and non-diarrheal cases

In total, 1121 diarrhea cases and 319 non-diarrheal cases were recruited into this study from June 2014 to July 2015. DEC was detected in 127 (11.33%) diarrhea cases and 9 (2.82%) controls (*χ*^2^ = 20.69, *P* < 0.001, *OR* = 4.36, 95% *CI*: 2.19–8.65), and the prevalence of NTS isolated from cases was higher than that of the controls across all age groups (*n* = 42, 3.75%, *n* = 1, 0.31%, *χ*^2^ = 10.10, *P* = 0.002, *OR* = 12.38, 95% *CI*: 1.70–90.29). In diarrhea cases, EPEC (5.44%, *n* = 61) was the most common subtype of DEC, followed by EAEC (5.35%, *n* = 60), EIEC (0.45%, *n* = 5) and ETEC (0.09%, *n* = 1). In addition, *S. enteritidis* (1.87%, *n* = 21) was the most common subtype of NTS in cases, followed by *S. enterica* Typhimurium (1.07%, *n* = 12).

### Single antibiotic resistance of DEC and NTS strains

Many of the enteric bacterial pathogens isolated from diarrhea cases were widely resistant to antibiotics in Kunming City (Table 2, Additional file 2). The rate of resistance to these ten antibiotics of DEC showed significant differences (*χ*^2^ = 386.77, *P* < 0.001). The resistance of DEC to AMP, NAL, TCY, REP and SXT was highly prevalent (more than 50.00%, respectively, Table 2) among isolates, but the resistance rate of DEC to CEP, CTX and CIP was lower (less than 30.00%, respectively, Table 2). The resistance profiles differed among different serotypes of DEC. The rate of resistance to these ten antibiotics of EAEC, EPEC and EIEC are significantly different (*χ*^2^ = 191.18, *P* < 0.001; *χ*^2^ = 191.95, *P* < 0.001; *χ*^2^ = 18.84, *P* = 0.026, respectively). Resistance to AMP, NAL, TCY, REP and SXT was also highly prevalent (more than 40.00%, respectively, Table 2) among EAEC, EPEC and EIEC, but the resistance to CEP, CTX and CIP was also lower among EAEC and EPEC. The resistance rates of the subtypes of DEC to NAL were significantly different (*P* < 0.037), but the resistance rates of the subtypes of DEC to AMP, AMC, CEP, CTX, GEN, CIP, TCY, REP and SXT were not significantly different.

**Table 2:** The resistance of subtype of diarrheagenic *Escherichia coli* and non-typhoidal *Salmonella* isolated from diarrhea cases

Antibiotics	Classification	DEC (*n* = 127)					NTS (*n* = 42)			
		DEC *n* = 127 *n* (%)	EAEC *n* = 60 *n* (%)	EPEC *n* = 61 *n* (%)	EIEC *n* = 5 *n* (%)	ETEC *n* = 1 *n* (%)	NTS *n* = 42 *n* (%)	*Salmonella enteritidis n* = 21 n (%)	*Salmonella enterica* Typhimurium *n* = 12 *n* (%)	Other *Salmonella n* = 9 *n* (%)
AMP	Resistant	100 (78.74)	49 (81.67)	46 (75.41)	4 (80.00)	1 (100.00)	32 (76.19)	21 (100.00)	8 (66.67)	3 (33.33)
	Intermediate	3 (2.36)	1 (1.67)	1 (1.64)	1 (20.00)	0 (0.00)	0 (0.00)	0 (0.00)	0 (0.00)	0 (0.00)
	Susceptible	24 (18.90)	10 (16.67)	14 (22.95)	0 (0.00)	0 (0.00)	10 (23.81)	0 (0.00)	4 (33.33)	6 (66.67)
AMC	Resistant	47 (37.01)	19 (31.67)	26 (42.62)	2 (40.00)	0 (0.00)	6 (14.29)	6 (28.57)	0 (0.00)	0 (0.00)
	Intermediate	10 (7.87)	7 (11.67)	3 (4.92)	0 (0.00)	0 (0.00)	0 (0.00)	0 (0.00)	0 (0.00)	0 (0.00)
	Susceptible	70 (55.12)	34 (56.67)	32 (52.46)	3 (60.00)	1 (100.00)	36 (85.71)	15 (71.43)	12 (100.00)	9 (100.00)
CEP	Resistant	38 (29.92)	18 (30.00)	17 (27.87)	3 (60.00)	0 (0.00)	5 (11.90)	4 (19.05)	1 (8.33)	0 (0.00)
	Intermediate	11 (8.66)	8 (13.33)	3 (4.92)	0 (0.00)	0 (0.00)	4 (9.52)	2 (9.52)	2 (16.67)	0 (0.00)
	Susceptible	78 (61.42)	34 (56.67)	41 (67.21)	2 (40.00)	1 (100.00)	33 (78.57)	15 (71.43)	9 (75.00)	9 (100.00)
CTX	Resistant	23 (18.11)	9 (15.00)	12 (19.67)	2 (40.00)	0 (0.00)	4 (9.52)	4 (19.05)	0 (0.00)	0 (0.00)
	Intermediate	4 (3.15)	2 (3.33)	2 (3.28)	0 (0.00)	0 (0.00)	0 (0.00)	0 (0.00)	0 (0.00)	0 (0.00)
	Susceptible	100 (78.74)	49 (81.67)	47 (77.05)	3 (60.00)	1 (100.00)	38 (90.48)	17 (80.95)	12 (100.00)	9 (100.00)
GEN	Resistant	39 (30.71)	25 (41.67)	13 (21.31)	1 (20.00)	0 (0.00)	5 (11.90)	2 (9.52)	2 (16.67)	1 (11.11)
	Intermediate	3 (2.36)	2 (3.33)	1 (1.64)	0 (0.00)	0 (0.00)	0 (0.00)	0 (0.00)	0 (0.00)	0 (0.00)
	Susceptible	85 (66.93)	33 (55.00)	47 (77.05)	4 (80.00)	1 (100.00)	37 (88.10)	19 (90.48)	10 (83.33)	8 (88.89)
NAL	Resistant	65 (51.18)	38 (63.33)	25 (40.98)	2 (40.00)	0 (0.00)	28 (66.67)	19 (90.48)	7 (58.33)	2 (22.22)
	Intermediate	7 (5.51)	1 (1.67)	5 (8.20)	0 (0.00)	1 (100.00)	4 (9.52)	2 (9.52)	2 (16.67)	0 (0.00)
	Susceptible	55 (43.31)	21 (35.00)	31 (50.82)	3 (60.00)	0 (0.00)	10 (23.81)	0 (0.00)	3 (25.00)	7 (77.78)
CIP	Resistant	7 (5.51)	4 (6.67)	3 (4.92)	0 (0.00)	0 (0.00)	0 (0.00)	0 (0.00)	0 (0.00)	0 (0.00)
	Intermediate	7 (5.51)	1 (1.67)	4 (6.56)	2 (40.00)	0 (0.00)	4 (9.52)	1 (4.76)	2 (16.67)	1 (11.11)
	Susceptible	113 (88.98)	55 (91.67)	54 (88.52)	3 (60.00)	1 (100.00)	38 (90.84)	20 (95.24)	10 (83.33)	8 (88.89)
TCY	Resistant	88 (69.29)	36 (60.00)	48 (78.69)	3 (60.00)	1 (100.00)	21 (50.00)	10 (47.62)	9 (75.00)	2 (22.22)
	Intermediate	0 (0.00)	0 (0.00)	0 (0.00)	0 (0.00)	0 (0.00)	0 (0.00)	0 (0.00)	0 (0.00)	0 (0.00)
	Susceptible	39 (30.71)	24 (40.00)	13 (21.31)	2 (40.00)	0 (0.00)	21 (50.00)	11 (52.38)	3 (25.00)	7 (77.78)
REP	Resistant	124 (97.64)	60 (100.00)	58 (95.08)	5 (100.00)	1 (100.00)	42 (100.00)	21 (100.00)	12 (100.00)	9 (100.00)
	Intermediate	1 (0.79)	0 (0.00)	1 (1.64)	0 (0.00)	0 (0.00)	0 (0.00)	0 (0.00)	0 (0.00)	0 (0.00)
	Susceptible	2 (1.57)	0 (0.00)	2 (3.28)	0 (0.00)	0 (0.00)	0 (0.00)	0 (0.00)	0 (0.00)	0 (0.00)
SXT	Resistant	79 (62.20)	40 (66.67)	35 (57.38)	3 (60.00)	1 (100.00)	11 (26.19)	6 (28.57)	3 (25.00)	2 (22.22)
	Intermediate	0 (0.00)	0 (0.00)	0 (0.00)	0 (0.00)	0 (0.00)	5 (11.90)	3 (14.29)	2 (16.67)	0 (0.00)
	Susceptible	48 (37.80)	20 (33.33)	26 (42.62)	2 (40.00)	0 (0.00)	26 (61.90)	12 (57.14)	7 (58.33)	7 (77.78)

Notes: *AMP* Ampicillin, *AMC* Amoxicillin and Clavulafiate, *CEP* Cephalothin, *CIP* Ciprofloxacin, *CTX* Cefotaxime, *DEC* Diarrheagenic *Escherichia coli, GEN* Gentamicin, *NAL* Nalidixic acid, *NTS* Non–typhoideal *Salmonella*, *REP* Rifampicin, *SXT* Sulfamethoxazole–Trimethoprim, *TCY* Tetracycline

DEC has been classified into several subtypes based on mechanisms of pathogenicity and clinical feature, such as enteropathogenic *E. coli* (EPEC), enterotoxigenic *E. coli* (ETEC), enteroaggregative *E. coli* (EAEC), enteroinvasive *E. coli* (EIEC), enterohemorrhagic *E. coli* (EHEC). NTS was discerned with serum agglutination test into *S. enteritidis*, *S. enterica* Typhimurium and other *Salmonella*

The rates of resistance to these ten antibiotics of NTS were significantly different (*χ*^2^ = 191.16, *P* < 0.001). Resistance to AMP, NAL, TCY and REP was also highly prevalent (more than 50.00%, respectively, Table 2) among isolates, but resistance to AMC, CEP, CTX, GEN and CIP was less prevalent. The resistance profiles differed among different serotypes of NTS, with the rates of resistance to these ten antibiotics of *S. enteritidis*, *S. enterica* Typhimurium and other *Salmonella* showing significant differences (*χ*^2^ = 113.12, *P* < 0.001; *χ*^2^ = 79.12, *P* < 0.001; *χ*^2^ = 46.44, *P* < 0.001, respectively). The resistance rates of *S. enteritidis* and *S. enterica* Typhimurium to AMP, NAL, TCY and REP was very serious (from 47.62% to 100.00%, Table 2), but to CEP, CTX, GEN and CIP was very low (from 0.00 to 19.05%). The resistance rates of the subtypes of NTS to AMP and NAL were significantly different (*P* < 0.001, equally), but the resistance rates of the subtypes of NTS to AMC, CEP, CTX, GEN, CIP, TCY, REP and SXT were not significantly different.

In diarrheal patients, the resistance rate of DEC and NTS strains to AMC (37.01% vs 14.29%, χ^2^ = 7.57, *P* = 0.006), CEP (29.92% vs 11.90%, *χ*^2^ = 5.40, *P* = 0.002), GEN (30.71% vs 11.90%, *χ*^2^ = 5.80, *P* = 0.016), TCY (69.29% vs 50.00%, *χ*^2^ = 5.13, *P* = 0.035) and SXT (62.20% vs 26.19%, *χ*^2^ = 16.44, *P* < 0.001) were significantly different. The resistance rate of DEC to AMC, CEP, GEN, TCY and SXT were more serious than the resistance rates of NTS to those antibiotics, but the resistance rate of DEC and NTS to AMP, CTX, NAL, CIP and REP was not significantly different. In diarrheal cases in patients less than 5 years of age, the resistance rate of DEC and NTS strains to AMC (35.48% vs 15.00%, *χ*^*2*^ = 5.66, *P* = 0.017), CEP (35.48% vs 12.50%, *χ*^2^ = 7.24, *P* = 0.007), GEN (31.18% vs 12.50%, *χ*^2^ = 5.13, *P* = 0.024), TCY (68.82% vs 50.00%, *χ*^2^ = 4.26, *P* = 0.039) and SXT (66.67% vs 27.50%, *χ*^2^ = 17.33, *P* < 0.0001) were significantly different, but there were no significant differences among the resistance rates of DEC and NTS to AMP, CTX, NAL, CIP and REP. In diarrheal patients over 5 years of age, the resistance rate of DEC and NTS to those ten antibiotics were not significantly different.

In diarrhea patients, the resistance rate of DEC isolated from cases under 5 years of age to AMP (83.87% vs 64.71%, *χ*^2^ = 5.46, *P* = 0.002), CEP (35.48% vs 14.71%, *χ*^2^ = 5.13, *P* = 0.024) and CTX (22.58% vs 5.88%, *χ*^2^ = 4.68, *P* = 0.03) were higher than those of the DEC strain isolated from patients over 5 years of age. The resistance rate of DEC isolated from cases under 5 years of age and over 5 years of age to AMC, GEN, NAL, CIP, TCY, REP and SXT were not significantly different. The resistance rate of NTS strains isolated from patients less than 5 years of age and over 5 years of age to these ten antibiotics were not significantly difference.

As shown in Table 3, in all diarrhea cases and controls across all age groups, the resistance rate for CIP of DEC strains isolated from diarrhea patients and controls showed significant differences (5.51% vs 33.33%, *P* = 0.018). For the other nine antibiotics, the resistance rate of DEC strains isolated from diarrheal cases and subjects were not significantly different. For subjects under 5 years, the resistance rate of DEC strains from diarrheal patients were not significantly different from those resistance rates of DEC strains isolated from controls. In subjects over 5 years, the resistance rate for CIP of DEC isolated from diarrhea patients was lower than that of controls (2.94% vs 75.00%, *P* = 0.002), and the resistance rates of DEC strains isolated from cases and controls for the other nine antibiotics were not significantly different.

**Table 3:** The comparison of the resistance of diarrheagenic *Escherichia coli* isolated from diarrhea cases and non–diarrhea subjects (age stratification)

Antibiotics	All age groups				< 5 years				≥ 5 years			
	Diarrhea *n* = 127 n (%)	Control *n* = 9 *n* (%)	*χ* ^2^	*P* value	Diarrhea *n* = 93 *n* (%)	Control *n* = 5 *n* (%)	*χ* ^2^	*P* value	Diarrhea *n* = 34 *n* (%)	Control *n* = 4 *n* (%)	*χ* ^2^	*P* value
AMP	100 (78.74)	8 (88.89)	–	0.294*	78 (83.87)	5 (100.00)	–	0.428*	22 (64.71)	3 (75.00)	–	0.405*
AMC	47 (37.01)	4 (44.44)	–	0.245*	33 (35.48)	1 (20.00)	–	0.318*	14 (41.18)	3 (75.00)	–	0.194*
CEP	38 (29.92)	4 (44.44)	–	0.184*	33 (35.48)	2 (40.00)	–	0.348*	5 (14.71)	2 (50.00)	–	0.132*
CTX	23 (18.11)	3 (33.33)	–	0.166*	21 (22.58)	2 (40.00)	–	0.252*	2 (5.88)	1 (25.00)	–	0.266*
GEN	39 (30.71)	5 (55.56)	–	0.091*	29 (31.18)	3 (60.00)	–	0.157*	10 (29.41)	2 (50.00)	–	0.291*
NAL	65 (51.18)	6 (66.67)	–	0.187*	52 (55.91)	3 (60.00)	–	0.349*	13 (38.24)	3 (75.00)	–	0.167*
CIP	7 (5.51)	3 (33.33)	–	0.018*	6 (6.45)	0 (0.00)	–	0.724*	1 (2.94)	3 (75.00)	–	0.002*
TCY	88 (69.29)	5 (55.56)	–	0.192*	64 (68.82)	3 (60.00)	–	0.328*	24 (70.59)	2 (50.00)	–	0.291*
REP	124 (97.64)	9 (100.00)	–	0.813*	91 (97.85)	5 (100.00)	–	0.900*	33 (97.06)	4 (100.00)	–	0.895*
SXT	79 (62.20)	5 (55.56)	–	0.250*	62 (66.67)	2 (40.00)	–	0.178*	17 (50.00)	3 (75.00)	–	0.278*

**Notes:** 1: *AMP* Ampicillin, *AMC*Amoxicillin and Clavulafiate, *CEP* Cephalothin, *CTX* Cefotaxime, *GEN* Gentamicin, *NAL* Nalidixic acid, *CIP* Ciprofloxacin, *TCY* Tetracycline, *REP* Rifampicin, *SXT* Sulfamethoxazole–Trimethoprim

2: The “–” means that the data cannot be calculated, the “*” means that the data was calculated with Fisher exact

3: The bold was showed there had significant difference between two groups

### Multidrug resistance of DEC and NTS strains

DEC isolates from diarrhea patients were resistant to only five (*n* = 22, 17.32%) or six (*n* = 22, 17.32%) of the most common antimicrobials, followed by only seven types of antimicrobials (*n* = 20, 15.75%) and only four types of antimicrobials *(n* = 17, 13.39%). No DEC strains from cases were resistant to ten types of antimicrobials. In addition, DEC strains isolated from controls showed relatively high resistance to only six types of antimicrobials (*n* = 4, 44.44%), followed by only nine types of antimicrobials (*n* = 2, 22.22%). Resistance to only one, two, seven or eight types of antimicrobials was not found. The resistance rate of DEC isolated from diarrhea cases to only nine types of antimicrobials was lower than that of DEC isolates from controls (1.57% vs 22.22%, *P* = 0.021), and the resistance rates to only one, two, three, four, five, six, seven, eight or ten types of antimicrobials were not significantly different for DEC isolated from diarrhea cases and controls. Resistance rates of DEC isolates from cases and controls to more than three types of antimicrobials were not significantly different (81.10% vs 88.89%, *P* = 0.33).

In diarrhea patients across all age groups, the resistance rate of DEC to only one type of antibiotic was lower than that of NTS to only one type of antibiotic (7.09% vs 16.67%, *P* = 0.047), and the resistance rate of DEC strains to only three antibiotics or four antibiotics were lower than those of NTS to only three antibiotics or four antibiotics (8.66% vs 19.05%, *χ*^2^ = 7.64, *P* = 0.043; 13.39% vs 33.33%, *χ*^2^ = 8.38, *P* = 0.004). For diarrhea cases under 5 years of age, the resistance rate of DEC strains to only one antibiotic or four antibiotics were all lower than those of NTS to only one antibiotic or four antibiotics (4.30% vs 14.71%, *P* = 0.046; 8.60% vs 26.47%, *P* = 0.01). The MDR resistance rate of NTS among diarrhea cases under 5 years of age and over 5 years of age were not significantly different.

### Molecular epidemiological characteristics of DEC and NTS strains

**Fig. 1:**
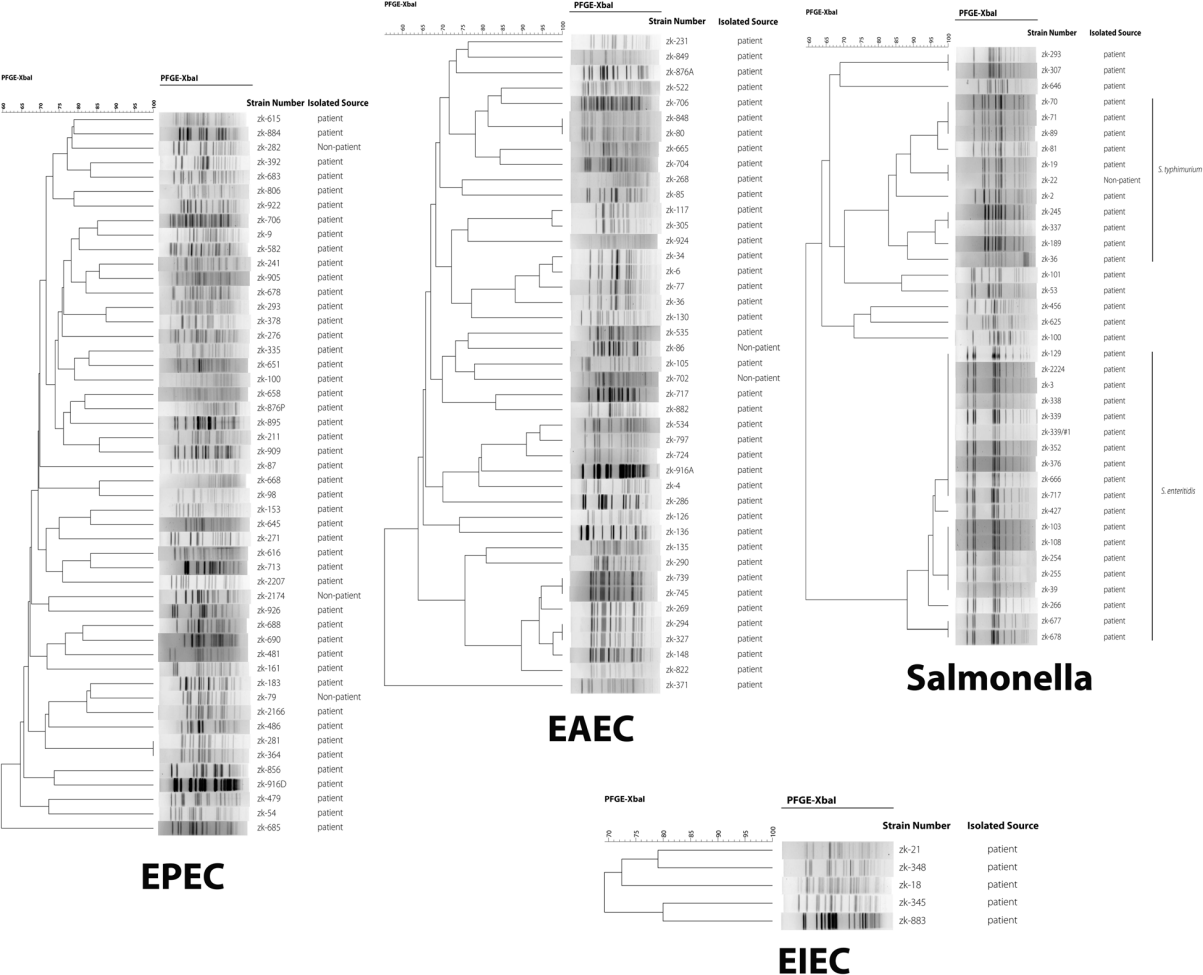
The molecular epidemiology of DEC and NTS strains isolated from subjects under five years of age. The PFGE pattern of DEC was discrete, but the pulsotype pattern of NTS strains were closely related to the serotype of NTS. The dendrogram was produced using the Dice coefficient and the UPGMA with a position tolerance of 1.0%; PFGE using XbaI endonuclease

**Fig. 2:**
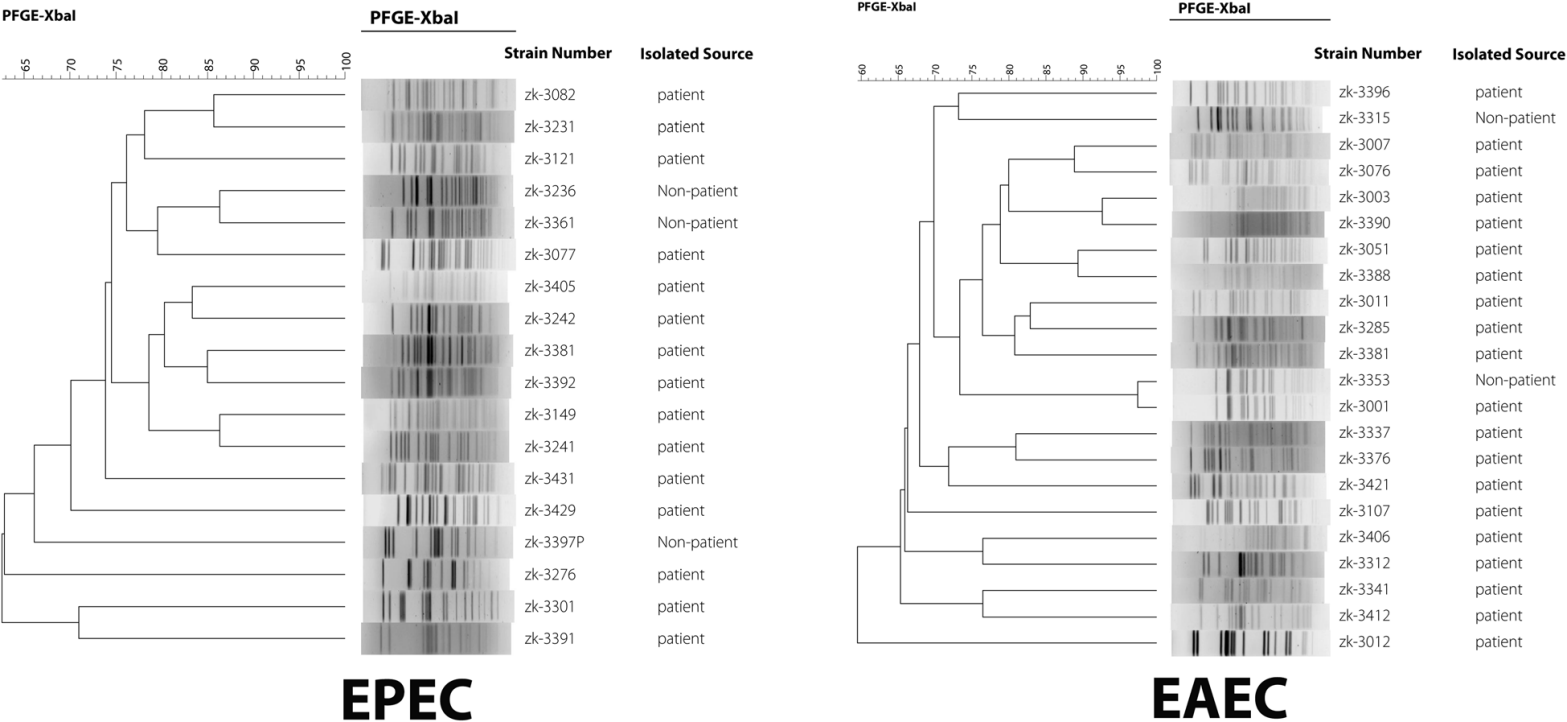
The molecular epidemiology of DEC and NTS strains isolated from subjects over five years of age. The PFGE pattern of DEC was discrete, but the pulsotype pattern of NTS strains was closely related to the serotype of NTS. The dendrogram was produced using the Dice coefficient and the UPGMA with a position tolerance of 1.0%; PFGE using XbaI endonuclease

PFGE was conducted to determine the clonal-relatedness among DEC and NTS strains. Pulsotype patterns of DEC strains had a high polymorphism and no identical profiles in any two DEC strains isolated from acute diarrhea cases and controls were found (Figs. 1 and 2). The pulsotype pattern of NTS strains was closely related to the serotype of NTS. The patterns of *S. Enteritis* and *S. enterica* Typhimurium showed two clusters. *S. enteritidis* had high similarity, but the pulsotype pattern of *S. enterica* Typhimurium strain was discrete (Figs. 1 and 2).

## Discussion

According to the WHO guidelines for the treatment of diarrhea, antimicrobials should not be used routinely, particularly for unknown causative agents [23]. Antimicrobials are often used as a supplement for children with bloody diarrhea. However, antimicrobials have been widely used to empirically treat diarrheal illness in China, because enteric pathogen identification and diagnosis is not feasible due to limited resources (in terms of funding and diagnostic techniques) [27]. Therefore, extensive knowledge of the prevalence of antibiotic resistance among the various bacterial species would be extremely valuable information.

Despite their benefits, antibiotics are now considered potentially harmful to individuals. For instance, the misuse of antibiotics was associated with microbiota impairment and related disorders [28]. Human micro-ecological factors play an important role in human nutrition, growth and development, biological antagonism and immunity. However, undesirable impacts have gradually increased with the widespread use of antimicrobial drugs, including flora imbalance, double infections and a decrease in host resistance to infection [29, 30].

### Single resistant strains are ubiquitous

Traditional antibiotics including NAL, SXT, AMP and TCY showed low activity against the DEC and NTS strains, which suggests that these four antibiotics should not be used as a first-line therapeutic drug for *Enterobacteriaceae*. The resistance to these four antibiotics was greater than 50.00%. This is higher than that found in other studies conducted in other developing countries [31, 32], but similar to previous studies conducted in China and other countries [15, 33, 34]. Although quinolones and fluoroquinolones were recommended as a first-line antimicrobial drug to treat diarrheal illness caused by *Enterobacteriaceae* [35], the first-generation fluoroquinolones (NAL) showed more serious resistance to strains in the study. This result was similar to other studies conducted in China [34, 36, 37], but was far higher than that found in Niger [32]. This high resistance rate was attributed to the misuse of NAL in China.

CIP showed antimicrobial activity against *Enterobacteriaceae* in this study, which was similar to results in other studies conducted in China and other countries [32, 38]. Unfortunately, CIP is poorly available due to weak profits in production and sales in China. Hence, doing away with the ‘drug price addition policy’ and the ‘drugs to support hospitals’ policy could enhance availability of inexpensive but effective antibiotics. The storage of low-price essential medicines will be establishment and popularization in China to some degree. These measures may be particularly important to reduce antibiotic resistance.

CTX is a third-generation cephalosporin used to treat illnesses involving gram-negative bacteria, and has been a popular empirical drug to treat severe gastrointestinal infection. The resistance rate of DEC (18.11%) and NTS (9.52%) to CTX is concerning, and should not be ignored. It may be that this resistance strain to third-generation cephalosporin produces ESBL, an enzyme that confers resistance to cephalosporin antibiotics and oxyimino-β-lactam synthetic drugs, as well as to other penicillin drugs. ESBL can be inhibited with clavulanic acid [39]. With infections caused by ESBL-producing *Enterobacteriaceae* increasing worldwide, treatment costs may substantially increase, and treatment periodicity may be prolonged. AMC was also sensitive to DEC and NTS strains in the study, suggesting that acute diarrheal disease caused by *Enterobacteriaceae* might be treated effectively with systemically united antibiotics. This would speed diarrhea recovery time and reduce medical fees, also lead lowering antibiotic resistance rates.

The resistance rates of DEC and NTS in this study were similar to those in a previous study in China [38], but were higher than that found in Africa [40]. To some extent, serious resistance may be attributed to antibiotic misuse in China. The overuse of antibiotics in China has seen the highest growth in world, with a large amount of antibiotics prescribed by doctors to both outpatients and inpatients. High expectations among patients to speed up symptom relief and recovery leads many patients to the erroneous belief that antibiotics are needed. Thus, the inappropriate administration of antibiotics has become more common, especially through intravenous infusion in patients with viral or parasitic infectious disease [41]. In addition, because antimicrobial prescription is a profit source for hospitals and doctors, further pressure is applied to doctors to prescribe more powerful antibiotics [42, 43]. At the same time, although the purchase of antibiotics from retail pharmacies without a prescription is forbidden by China’s Food and Drug Administration, over-the-counter sale of antimicrobials without a prescription is possible and may aggravate antibiotic resistance and spread resistant strains [12, 42]. Hence, widespread public health education and supervision of the sales and prescription of antibiotics in retail pharmacies and hospitals are urged [42, 44].

### The prevalence of multidrug-resistant strains is significant

In both diarrheal and non-diarrheal subjects, the MDR rates of DEC and NTS were over 75.00%, a finding similar to other studies [45, 46]. It was an unexpected finding that the drug-resistant strains were likely clonally related and ubiquitous in Kunming City, also implying that the resistant strains did not aggravate strain pathogenicity. In addition, the prevalence of the MDR strain may have arisen in China in the last 20 years. This may be due to the accumulation of resistant genes in a single-clone bacterial strain and/or the expression of genes that code for multidrug efflux pumps, extruding a series of antibiotics [47].

### Combining molecular characteristics and epidemiological investigation can aid in precise tracking of the source

The molecular pattern of DEC were highly polymorphic, as found in another study [48]. This may be attributed to the fact that the genome of DEC shows high plasticity, given that the epidemiologic investigations conducted on DEC-infected subjects showed no obvious epidemiological association between two DEC-infected individuals. This suggests that there was no obvious aggregation of diarrhea cases infected with DEC, and no outbreak of acute diarrhea episodes caused by DEC. Hence, PFGE alone was not sufficient to provide accurate evidence for tracking the infection source, and required the addition of epidemiological investigation. Patterns of *S. enteritidis* strains showed almost identical characteristics, but there was no connection between any two diarrhea cases based on epidemiological investigation. It can be concluded that *S. enteritidis* formed a dominant strain in local populations in recent years. Patterns of *S. enterica* Typhimurium strains showed discrete characteristics suggesting that the preponderant strain was not local, as seen in other studies [18, 49]. The combination of molecular patterns and epidemiological investigation was a more accurate method to explore diarrheal outbreaks caused by enteric bacterial pathogens and to track the source.

### Limitations of this study

Several limitations of this study can be noted. First, antibiotic resistance genes (ARGs) have important associations with strains of phenotypic resistance, but ARGs were not detected in this study. Hence, the relationship between ARGs and phenotypic resistance was not revealed. Second, the strains producing carbapenemases and ESBL were not detected, which was obstructing treatment to some extent. Third, the serotypes of the DEC strains were not detected, and thus the correlation between DEC serotypes and DEC pulsotype patterns were not clear. Therefore, further research including ARGs, phenotypic resistance and ESBL should be conducted in the future.

## Conclusions

Antibiotic resistance of bacterial strains was very significant, and multidrug-resistant strains were widely prevalent in diarrheal children. Hence, it is urgent to regulate antibiotic use. Combining the study of the molecular characteristics of enteric bacterial pathogens with systematic epidemiological investigation can provide accurate evidence to track the source of infections.

## Additional files

Additional file 1: Multilingual abstract in the five official working languages of the United Nations. (PDF 838 kb)

Additional file 2: Raw data on antimicrobial microbial resistances used in this work. (SAV 35 kb)

## Abbreviations

AMC: Amoxicillin and Clavulafiate
AMP: Ampicillin
APW: Alkaline peptone water
ARGs: Antibiotic resistance genes
CEP: Cephalothin
*CI*: Confidence interval
CIP: Ciprofloxacin
CLSI: Clinical and laboratory standards institute
Cm: Centimeter
CTX: Cefotaxime
DEC: Diarrheagenic *Escherichia coli*
EAEC: Enteroaggregative *Escherichia coli*
EHEC: Enterohemorrhagic *Escherichia coli*
EIEC: Enteroinvasive *Escherichia coli*
EPEC: Enteropathogenic *Escherichia coli*
ESBL: Extended spectrum β lactamases
ETEC: Enterotoxigenic *E. coli*
GEN: Gentamicin
I: Intermediate
MAC: Macconkey agar
MDR: Multi-drug resistance
NAL: Nalidixic acid
NTS: Non-typhoidal Salmonella
*OR*: Odds ratios
PFGE: Pulse field gel electrophoresis
qPCR: Quantitative PCR
R: Resistant
REP: Rifampicin
S: Susceptible
SBG: Selenite brilliant green sulfa enrichment broth
SS: Salmonella-Shigella agar
SXT: Sulfamethoxazole-Trimethoprim
TCBS: Thiosulfate-citrate-bile salts-sucrose
TCY: Tetracycline
UPGMA: Unweighted pair group methods with the arithmetic mean algorithm
WHO: World Health Organization
XLD: Xylose, lysine and deoxycholate agar
