# Supplementary material for: Antibiotic resistance and molecular characterization of diarrheagenic Escherichia coli and non-typhoidal Salmonella strains isolated from infections in Southwest China
Source: Infect Dis Poverty. 2018 May 7;7:53. doi: 10.1186/s40249-018-0427-2 (PMC5964730; doi:10.1186/s40249-018-0427-2)
Supplement: Supplementary file 1 — Multilingual abstract in the five official working languages of the United Nations. (PDF 838 kb) [file 40249_2018_427_MOESM1_ESM.pdf]

Translation of the abstract into the five official working languages of the United Nations

مقاومة المضادات الحيوية والخصائص الجزيئية للإشريكية القولونية *المثيرة للإسهال* والسالمونيلا *غير التيفية* في السلاسل المعزولة عن الإصابات بالعدوى في جنوب غرب الصين

قدمه: شون شيان تشانغ، ويونغ مينغ تشو، ولي قوانغ تيان، وجيا-شو تشن، وريتا تينوكو توريس، وإيمانويل سيرانو، وشي تشو لي، وشاو هونغ تشن، ولين عاي، وجون هو تشن، وشانغ شيا، ويان لو، وشان لف، وشيويه جياو تنغ، ووين شو، ووين بينغ قو، وسي تانغ جونج، وشياو نونغ تشو، ولان لان قنغ، ووي هو

الملخص

المعلومات الأساسية: يُعتبر الإسهال الجرثومي واحدًا من أكثر الأسباب الشائعة التي تستدعي الاستشارة الطبية، وتُسبب الوفيات والأمراض في العالم. إن الإشريكية القولونية المثيرة للإسهال (DEC) *والسالمونيلا* غير التيفية(NTS)هي من مسببات الأمراض المعوية الرئيسة في البلدان النامية، والاستخدام العشوائي للمضادات الحيوية أسهم على نحوٍ كبير في صعوبة مقاومتها. بالتالي، يكمن الغرض من هذه الدراسة في تحديد أنماط مقاومة مضادات الميكروبات والخواص الجزيئية للإشريكية القولونية المثيرة للإسهال والسالمونيلا غير التيفية في جنوب غرب الصين.

الطرق: جُمِعَ عدد ١١٢١ مريض مصاب بالإسهال و٣١٩ من المرضى المشاركين دون المصابين بالإسهال من شتى الفئات العمرية من أربعة مستشفيات راصدة للمرض منذ يونيو ٢٠١٤م وحتى يوليو ٢٠١٥م في مدينة كونمينغ، في مقاطعة يونان. جُمعت كل عينة من البراز بغرض رصد الإشريكية القولونية المثيرة للإسهال والسالمونيلا غير التيفية باستخدام الأساليب المايكروبيولوجية والجزيئية الموحدة. أُجريت فحوص مقاومة مضادات الميكروبات باستخدام طريقة نشر قرص بأور- كيربي، كماتتوافق المعايير المتعلقة بفحص قابلية مضادات الميكروبات مع معهد المعايير السريرية والمخبرية (CLSI). أُجريَ تحديد الصفات الجزيئية للسلاسلباستخدام تقنية الفصل الكهربي الهلامي بالحقل النبضي. أُستخدم استبيان منظم بعناية لتدوين البيانات الأساسية المتعلقة بالأوبئة (على سبيل المثال:الجنس، والعمر، ومكان الإقامة، والفصل، إلى آخره). أُجريَ تحليل البيانات باستخدام اختبار مربع كاي أو اختبار فيشر الدقيق.

النتائج: رُصِدَت الإشريكية القولونية المثيرة للإسهال بعدد ١٢٧ حالة (بمعدل ١١,٣٣٪) إصابة بالإسهال وبعدد ٩ حالات (بمعدل٢,٨٢٪) من دون المصابة بالإسهال (χ^2= ٢٠,٦٩، *القيمة الاحتمالية* < 0.001, أو = 4.36, 95% فاصل الثقة : 2.19–8.65)، وكان معدل مدى انتشار حالات السالمونيلا غير التيفية المعزولة عن حالات الإصابة بالإسهال مرتفعاً أكبر من ذلك للحالات غير المصابة بالإسهال من مختلف الفئات العمرية (العدد = 42, 3.75%, العدد = 1, 0.31 %, χ^2= 10.10, *القيمة الاحتمالية* = 0.002, أو = 12.38, 95% فاصل الثقة : 1.70–90.29). أظهرت معدلات مقاومة عشر مضادات لميكروبات الإشريكية القولونية المثيرة للإسهال والسالمونيلا غير التيفية تباينات بقدرٍ كبير (χ^2= 386.77, القيمة الاحتمالية < 0.001; χ^2= 191.16، القيمة الاحتمالية < 0.001). كانت معدلات المقاومة للأموكسيسللين والكلافيولينات (AMC), والسيفالوثين (CEP), والجنتاميسين (GEN) ومضاد التريميثوبريم/سلفاميثوكسازول لحالات الإشريكية القولونية المثيرة للإسهال المعزولة عن حالات الإصابة بالإسهال مرتفعة أكثر من تلك الحالات للسالمونيلا غير التيفية من المرضى المصابين بالإسهال (37.01% vs 14.29%, χ^2 = 7.57, القيمة الاحتمالية= 0.006; 29.92% مقابل 11.90%, χ^2= 5.40, القيمة الاحتمالية= 0.02; 37.01% مقابل 11.90%, χ^2 = 5.80, القيمة الاحتمالية= 0.016; 62.20% مقابل 26.19%, χ^2= 16.44, القيمة الاحتمالية < 0.001; على التوالي). كان مضاد السيبروفلوكساسين المضاد الحيوي الأكثر حساسية لسلاسل الإشريكية القولونية المثيرة للإسهال والسالمونيلا غير التيفية المعزولة عن حالات الإصابة بالإسهال. لم تُظهر معدلات مقاومة الإشريكية القولونية المثيرة للإسهال المعزولة عن الحالات والتي تُسيطر على أكثر من ثلاثة أنواع من مضادات الميكروبات (مقاومة الأدوية المتعددة) أي تباينات كبيرة (81.10% مقابل 88.89%, القيمة الاحتمالية= 0.33). كانت أنماط السلاسل البكتيرية المفصولة لسلاسل الإشريكية القولونية المثيرة للإسهال شديدة التنوع; ولكن، كان نمط السلاسل البكتيرية المفصولة لسلاسل السالمونيلا غير التيفية مرتبطارتباطاً وثيقاً بالنمط المصلي. كان نمط سالمونيلا كوليرا الخنازير شبيهاً بدرجة عالية، إلا أن سلسلة السالمونيلا التيفية *الفأرية*كانت منفصلة.

الاستنتاجات : تُثيرُ مقاومة المضادات الحيوية *للبكتيريا المعوية* قلقاً كبيرًا. تُبرر الآثار الاجتماعية المترتبة على استخدام المضادات الحيوية الحاجة للرقابة الصارمة لمحاربة ازدياد معدل مقاومة مضادات الميكروبات. يمكن أن يُقدم الاستقصاء في علم الأوبئة الجزيئي وعلم الوبائيات الممنهج أدلة دقيقة لتتبع مصدر العدوى.

Translated from English version into Arabic by Bashaier Allam, proofread by Maher Souilah, through

中国西南地区致腹泻性大肠杆菌和非典型性沙门菌的耐药和分子流行病学特征研究

张顺先，周永明，田利光，陈家旭，Rita Tinoco-Torres，Emmanuel Serrano，李石柱，陈韶红，艾琳，陈军虎，夏尚，卢艳，吕山，滕雪娇，徐闻，古文鹏，龚四堂，周晓农，耿岚岚，胡薇

摘要：

引言：细菌性腹泻是引起全球人群就诊、发病和死亡的最常见原因之一。致病性大肠埃希菌和非伤寒沙门氏菌是很多发展中国家的导致腹泻的细菌性病原体，因此抗生素的滥用极大地促进了这些菌株的耐药性。因此，本研究的目的是揭示中国西南地区致腹泻大肠杆菌和非典型性沙门菌的耐药特征和分子流行病学特征。

方法：从 2014 年 7 月 1 号到 2015 年 6 月 30 号，在云南省昆明市选择 4 家哨点医院作为研究现场，共纳入 1121 名急性腹泻患者和 319 名非腹泻研究对象，收集粪便样本，使用标准的微生物检测方法检测粪便样本中的致病性大肠埃希菌和非伤寒沙门氏菌。抗菌素耐药实验采用纸片扩散法，参考标准采用美国临床实验室标准化协会的标准。菌株的分子流行病学的分析采用脉冲场凝胶电泳的方法。同时，采用结构化问卷收集研究对象的流行病学资料（如性别、年龄、居住地、季节等）。数据分析采用卡方检验或 Fisher 精确检验法。

结果：致腹泻性大肠杆菌在腹泻人群中检出 127 株（11.33%），在腹泻人群中检出 9 株（2.82%，$\chi^2 = 20.69$，$P < 0.001$, $OR = 4.36$，95% CI: 2.19–8.65）。非典型性沙门菌的检出率在腹泻人群中高于非腹泻人群（$n = 42$，3.75%，$n = 1$，0.31 %，$\chi^2 = 10.10$，$P = 0.002$，$OR = 12.38$, 95% CI：1.70–90.29）。致腹泻大肠杆菌和非典型性沙门菌对这十种抗生素的耐药率不完全相同（$\chi^2 = 386.77$，$P < 0.001$；$\chi^2 = 191.16$，$P < 0.001$）。在全年龄组腹泻人群中，致腹泻性大肠杆菌对阿莫西林克拉维酸、头孢噻吩、庆大霉素、磺胺甲基异恶唑-甲氧苄氨嘧啶的耐药率超过非典型性沙门菌对这些抗生素的耐药率（37.01% vs 14.29%，$\chi^2 = 7.57$，$P = 0.006$；29.92% vs 11.90%，$\chi^2 = 5.40$，$P = 0.02$；37.01% vs 11.90%，$\chi^2 = 5.80$，$P = 0.016$；62.20% vs. 26.19%，$\chi^2 = 16.44$，$P < 0.001$）。在腹泻人群中分离到的菌株的耐药实验中，环丙沙星对致腹泻性大肠杆菌和非典型性沙门菌都很是最敏感的。在腹泻人群和非腹泻人群中分离到的致腹泻性大肠杆菌中，其多重耐药没有差异（81.10% vs 88.89%，$P = 0.33$）。致腹泻性大肠杆菌的分子模型有很大的可塑性，呈现高度的可变性；而非典型性沙门菌的分子模型和其血清型有很大的关系，即肠炎沙门菌的分子模型很相似，但副伤害沙门菌的分子模型十分的离散。

结论：肠杆菌科细菌的抗生素耐药应该受到极大关注。全社会都应该动员起来，严格限制抗生素的使用。分子流行病学和系统的流行病学调查结合起来可以准确的追踪细菌性传染源。

Translated from English version into Chinese by Shun-Xian Zhang

Antibiorésistance et caractérisation moléculaire des souches entéropathogènes d'*Escherichia coli*

et des souches non typhiques de*Salmonella* isolées chez des patients infectés dans le sud-ouest de la Chine

Shun-Xian Zhang, Zhou Yong-Ming, Li-Guang Tian, Jia-Xu Chen, Rita Tinoco-Torres, Emmanuel Serrano, Shi-Zhu Li, Shao-Hong Chen, Lin Ai, Chen Jun-Hu, Shang Xia, Lu Yan, Shan Lv, Xue-Jiao Teng, Wen Xu, Gu Wen-Peng, Gong Si-Tang, Zhou Xiao-Nong, Lan-Lan Geng, Wei Hu

Résumé

Contexte: La diarrhée bactérienne est l'une des causes de consultations médicales, de mortalité et de morbidité les plus fréquentes au monde. Les souches d'*Escherichia coli* entéropathogènes (ECEP) et de *Salmonella* non typhiques (SNT) sont des pathogènes intestinaux importants dans les pays en voie de développement et l'utilisation excessive des antibiotiques a grandement contribué à l'apparition de souches résistantes. Le but de la présente étude est par conséquent de repérer les schémas d'antibiorésistance et les caractéristiques moléculaires des ECEP et des SNT dans le sud-ouest de la Chine.

Méthodes: 1121 patients atteints de diarrhée et 319 sujets sans diarrhée de tous les groupes d'âge ont été recrutés entre juin 2014 et juillet 2015 dans quatre hôpitaux sentinelles de la ville de Kunming, dans la province du Yunnan. Chaque échantillon de selles recueilli a été testé dans le but de détecter les ECEP et les SNT à l'aide de méthodes microbiologiques et moléculaires standard. La résistance aux antimicrobiens a été testée par la méthode de diffusion sur disques de Kirby-Bauer, dans le respect des normes relatives aux antibiogrammes du CLSI (Clinical and Laboratory Standards Institute). La caractérisation moléculaire des souches a été effectuée par électrophorèse sur gel en champ pulsé (PFGE). Un questionnaire structuré a été utilisé pour relever les données épidémiologiques de base : sexe, âge, lieu de résidence, saison, etc. Les données ont été analysées à l'aide du test du khi-deux ou du test exact de Fisher.

Résultats: Des ECEP ont été détectées chez 127 patients atteints de diarrhée (11,33 %) et chez 9 sujets sans diarrhée (2,82 %) ($\chi^2 = 20,69$, $P < 0,001$, $RC = 4,36$, *IC*à 95 % : de 2,19 à 8,65), et la prévalence des SNT isolées chez des patients atteints de diarrhée était plus élevée que celle des sujets sans diarrhée dans tous les groupes d'âge ($n = 42$, 3,75 %, $n = 1$, 0,31 %, $\chi^2 = 10,10$, $P = 0,002$, $RC = 12,38$, *IC* à 95 % : de 1,70 à 90,29). Les taux de résistance des ECEP et des SNT à dix antibiotiques ont révélé des différences significatives ($\chi^2 = 386,77$, $P < 0,001$; $\chi^2 = 191,16$, $P < 0,001$). Les taux de résistance à l'amoxicilline-acide clavulanique (AMC), à la céfalotine (CEF), à la gentamicine (GEN) et au triméthoprime-sulfaméthoxazole (SXT) des ECEP isolées sur des patients atteints de diarrhée étaient plus élevés que ceux des SNT isolées sur des patients atteints de diarrhée (37,01 % vs 14,29 %, $\chi^2 = 7,57$, $P = 0,006$; 29,92 % vs 11,90 %, $\chi^2 = 5,40$, $P = 0,02$; 37,01 % vs 11,90 %, $\chi^2 = 5,80$, $P = 0,016$; 62,20 % vs 26,19 %, $\chi^2 = 16,44$, $P < 0,001$, respectivement). La ciprofloxacine (CIP) s'est avérée l'antibiotique auquel les ECEP et les souches de SNT isolées sur des patients atteints de diarrhée étaient les plus sensibles. Les taux de résistance à plus de trois antibiotiques (multirésistance) n'ont pas été significativement différents entre les ECEP isolées sur des patients atteints de diarrhée et sur des témoins (81,10 % vs 88,89 %,$P = 0,33$). Les schémas PFGE des souches d'ECEP étaient très divers, tandis que ceux des souches de SNT présentaient une forte corrélation avec le sérotype. Le schéma de *S. enteritidis* était très similaire mais la souche *S. enterica* Typhimurium était à part.

Conclusions: La résistance aux antibiotiques des *Enterobacteriaceae* est un problème très préoccupant. Les effets au niveau sociétal de l'utilisation d'antibiotiques justifient la surveillance stricte de leur usage afin de lutter contre l'augmentation des antibiorésistances. L'épidémiologie moléculaire et des investigations épidémiologiques systématiques peuvent fournir des données exactes permettant de remonter à la source de l'infection.

Translated from English version into French by Suzane Assenat, proofread by Charlote Giovan, through

TRANSLATORS WITHOUT BORDERS

Антибиотикорезистентность и молекулярная характеристика диареегенной кишечной *палочки Escherichia coli* и нетифоидных *штаммов* Salmonella*, выделенных* из инфекций на юго-западе Китая

Шунсиань Чжан, Юнмин Чжоу, Лигуан Тянь, Цзясюй Чен, Рита Тиноко-Торрес, Эммануэль Серрано, Шичжу Ли, , Шаохун Чэнь, Лин Ай, Цзюньху Чэнь, Шан Ся, Янь Лу, Шань Лу, Сюэцзяо Тэн, Вэнь Сюй, Вэньпэн Гу, Ситан Гун, Сяонун Чжоу, Ланьлань Гэн, Вэй Ху

Аннотация

Исходные данные: Бактериальная диарея является одной из самых частых причин медицинских консультаций, смертности и заболеваемости в мире. Диареегенная кишечная*палочка Escherichia coli* (DEC) и нетифоиднаяSalmonella(NTS) – основные кишечные патогены в развивающихся странах, и неосмотрительное использование антибиотиков значительно способствовало появлению резистентных штаммов. Поэтому, цель текущей работы заключается в определении специфики противомикробной резистентности и молекулярных характеристик DEC и NTS в юго-западных регионах Китая.

Методы: 1121 пациентов с диареей и 319 участников исследования, не страдающих этим расстройством, всех возрастных групп были набраны из четырех дозорных больниц в период с июня 2014 года по июль 2015 года в городе Куньмин, провинция Юньнань. Каждый образец стула собирали для обнаружения DEC и NTS путём стандартных микробиологических и молекулярных методов. Тестирование противомикробной резистентности проводилось с использованием диско-диффузионного метода Кирби-Бауэра на чувствительность бактерий к антибиотикам, стандарты для тестирования антимикробной чувствительности соответствовали требованиям Института клинических и лабораторных стандартов(CLSI). Молекулярную характеристику штаммов проводили с использованием импульсного гель-электрофореза (PFGE). Для регистрации основных эпидемиологических данных (например, пола, возраста, места жительства, сезона и т. д.) использовался структурированный опросник. Данные анализировали с использованием критерия хи-квадрата или точного критерия Фишера.

Результаты: DEC была выявлена в 127 (11,33%) случаях диареи и в 9 (2,82%) недиарейных случаях (χ^{x2} = 20,69, *P* < 0,001, *OR* = 4,36, 95% *CI*: 2,19–8,65), и распространенность NTS,

выделенная из случаев диареи, была выше, чем распространенность в недиарейных случаях во всех возрастных группах (n = 42, 3,75%, n = 1, 0,31 %,χ^{x2} = 10,10, P = 0,002, OR = 12,38, 95% CI: 1,70–90,29). Показатели устойчивости DEC и NTS к десяти антибиотикам показали значительные различия (χ^2= 386,77, P< 0,001;χ^2 = 191,16, P < 0,001). Показатели устойчивости DEC к амоксициллину и клавулафиату (AMC), цефалотину (CEP), гентамицину (GEN) и сульфаметоксазол-триметоприму (SXT), выделенные из случаев диареи, были выше, чем у NTS, выделенных у пациентов с диареей (37,01% против 14,29%,χ^2 = 7,57,P = 0,006; 29,92% против 11,90%, χ^2= 5,40, P = 0,02; 37,01% против 11,90%, χ^2= 5,80, P = 0,016; 62,20% против 26,19%,χ^2 = 16,44,P < 0,001; соответственно).Ципрофлоксацин (CIP) был самым чувствительным антибиотиком для штаммов DEC и NTS, выделенных из случаев диареи. Показатели резистентности изолятов DEC пациентов с диареей и контрольных групп к более чем трём видам противомикробных препаратов (множественная лекарственная устойчивость, MDR) не выявили существенных различий (81,10% против 88,89%, P = 0,33). Пульсотипические структуры штаммов DEC были очень разнообразными; однако, пульсотипическая структура штаммов NTS была тесно связана с серотипом. Структура *S. Enteritidis* была очень похожа, но штамм *S. enterica* Typhimurium был обособлен.

Заключение: Сопротивление *Enterobacteriaceae* антибиотикам, это серьёзная проблема. Социальные последствия использования антибиотиков оправдывают строгий контроль противодействия увеличения резистентности противомикробным препаратам. Молекулярная эпидемиология и систематические эпидемиологические исследования могут предоставить достоверные сведения для определения источника инфицирования.

Translated from English version into Russian by Kate Kazyk, proofread by Sam Abramov, through

TRANSLATORS
WITHOUT BORDERS

Resistencia a los antibióticos y caracterización molecular de cepas diarreigénicas de *Escherichia coli* y de *Salmonella* no tifoidea aisladas de infecciones en China del Suroeste

Shun-Xian Zhang, Yong-Ming Zhou, Li-Guang Tian, Jia-Xu Chen, Rita Tinoco-Torres, Emmanuel Serrano, Shi-Zhu Li, Shao-Hong Chen, Lin Ai, Jun-Hu Chen, Shang Xia, Yan Lu, Shan Lv, Xue-Jiao Teng, Wen Xu, Wen-Peng Gu, Si-Tang Gong, Xiao-Nong Zhou, Lan-Lan Geng, Wei Hu

Resumen

Contexto: La diarrea bacteriana es una de las causas más frecuentes de consultas médicas, mortalidad y morbilidad en el mundo. La *Escherichia coli* diarreigénica (ECD) y la *Salmonella* no tifoidea (SNT) son agentes patógenos intestinales muy frecuentes en los países en desarrollo y el uso indiscriminado de antibióticos ha contribuido en gran medida a que las cepas se vuelvan resistentes. Así pues, el objetivo del presente estudio consiste en identificar los patrones de la resistencia a los antimicrobianos y las características moleculares de la ECD y la SNT en China del Suroeste.

Método: Se seleccionó un total de 1121 pacientes diarreicos y 319 sujetos no diarreicos de cuatro hospitales centinela entre junio de 2014 y julio de 2015 en la ciudad de Kunming, provincia de Yunnan. Se recogieron muestras de heces para detectar ECD y SNT a través de métodos microbiológicos y moleculares estándar. Se realizaron pruebas de resistencia a los antimicrobianos con el método de difusión en disco de Kirby-Bauer y las normas de las pruebas de susceptibilidad antimicrobiana cumplían las del Instituto de Normas Clínicas y de Laboratorio (CLSI). La caracterización molecular de cepas se llevó a cabo usando electroforesis en gel de campo pulsado (EGCP). Se realizó un cuestionario estructurado para registrar datos epidemiológicos básicos (p. ej. sexo, edad, lugar de residencia, estación, etc.). Los datos se analizaron mediante la técnica de la prueba de chi-cuadrado o la prueba exacta de Fisher.

Resultados: Se detectó ECD en 127 (11.33 %) casos diarreicos y en 9 (2.82 %) casos no diarreicos ($\chi^2 = 20.69$, $P < 0.001$, $O = 4.36$, 95 % *CI*: 2.19–8.65), y la prevalencia de aislamiento de SNT de casos diarreicos era más alta que la de casos no diarreicos en todas las cohortes de edad ($n = 42$, 3.75 %, $n = 1$, 0.31 %, $\chi^2 = 10.10$, $P = 0.002$, $OR = 12.38$, 95 % *CI*: 1.70–90.29). Las tasas de resistencia a diez antibióticos contra ECD y SNT mostraron diferencias importantes ($\chi^2 = 386.77$, $P < 0.001$; $\chi^2 = 191.16$, $P < 0.001$). Las tasas de resistencia a la amoxicilina y clavulanato (AMC), cefalothina (CEP), gentamicina (GEN) y sulfametoxazol y trimetoprima (SXT) de aislamiento de ECD de casos diarreicos eran más altas que las de aislamiento de SNT de pacientes diarreicos (37.01 % vs. 14.29 %, $\chi^2 = 7.57$, $P = 0.006$; 29.92 % vs. 11.90 %, $\chi^2 = 5.40$, $P = 0.02$; 37.01 % vs. 11.90 %, $\chi^2 = 5.80$, $P = 0.016$; 62.20 % vs. 26.19 %, $\chi^2 = 16.44$, $P < 0.001$; respectivamente). Laciprofloxacina (CIP) demostró ser el antibiótico más sensible para las cepas de ECD y SNT aisladas de casos diarreicos. Las tasas de resistencia de los aislamientos de ECD de casos y controles a más de tres tipos de antimicrobianos (resistencia a múltiples fármacos, RMF) no mostraron diferencias significativas (81.10 % vs. 88.89 %, $PAG = 0.33$). Los patrones de pulso de las cepas de ECD fueron muy diversos; sin embargo, el patrón de pulso de las cepas de SNT estaba estrechamente relacionado con el serotipo. El patrón de *S. enteritidis* fue muy similar, pero la cepa *S. enterica* Typhimurium fue más discreta.

Conclusiones: La resistencia de las *Enterobacteriaceae* a los antibióticos es muy preocupante. Los efectos sociales del uso de antibióticos justifican un control estricto para combatir el aumento de la resistencia a los antimicrobianos. La investigación epidemiológica molecular y la investigación epidemiológica sistemática pueden ofrecer señales bastante precisas para rastrear la fuente de infección.

Translated from English version into Spanish by Marr, proofread by Sara Fairen, through

TRANSLATORS WITHOUT BORDERS
